# A 1.5-GS/s 12-bit 58.9-dB SNDR Pipelined ADC with Weight-Regulated Dither-Based Interstage Gain Error Calibration

**DOI:** 10.3390/s26185899

**Published:** 2026-09-17

**Authors:** Shang Xu, Zheng Zhang, Daolin Zhang, Guoan Wu, Lamin Zhan

**Affiliations:** School of Integrated Circuits, Huazhong University of Science and Technology, Wuhan 430074, China; shangxu0214@hust.edu.cn (S.X.); zhangzhenga@hust.edu.cn (Z.Z.); dorian_201@hust.edu.cn (D.Z.); wuga5612@hust.edu.cn (G.W.)

**Keywords:** pipelined analog-to-digital converter, residue amplifier, interstage gain errors (IGE), background calibration, dither, least mean squares (LMS)

## Abstract

This paper presents a novel weight-regulated, dither-based hybrid dual-stage background calibration technique for interstage gain errors (IGEs) in pipelined analog-to-digital converters (ADCs). To evaluate IGEs, a 1-bit pseudorandom dither is first injected into the first three stages. The proposed algorithm then evaluates IGEs via correlation. The first stage employs a large fixed-step Least Mean Squares (LMS) algorithm to rapidly estimate the gain error. The second stage utilizes the first stage’s output to suppress fluctuations using a weighted error and refines the estimate through a rational-quadratic adaptive-variable-step (RQ-AVS) LMS method. The proposed technique was verified using a fabricated 1.5-GS/s 12-bit pipelined ADC prototype implemented in a 28-nm CMOS process and featuring a 5.9-GHz input bandwidth. Measurement results show that at a 745 MHz input, the signal-to-noise-and-distortion ratio (SNDR) and spurious-free dynamic range (SFDR) improve from 52.1 dB and 65.8 dBc to 58.9 dB and 74.3 dBc, respectively. During calibration, the algorithm converges within 0.12 million samples, reducing steady-state estimation variation from +10.93%/−12.51% to +1.29%/−2.13%. The ADC achieves +0.25/−0.24 LSB differential nonlinearity (DNL) and +0.80/−0.75 LSB integral nonlinearity (INL) with a power consumption of 102.4 mW.

## 1. Introduction

High-speed analog-to-digital converters (ADCs) are essential in broadband wireless receivers, high-speed data-acquisition systems, and wideband sensing platforms. These applications require high sampling rates, adequate resolution, and good linearity. For example, Wi-Fi 7 supports channel bandwidths of up to 320 MHz and 4096-quadrature amplitude modulation (4096-QAM). Direct digitization of such a channel requires a sampling rate of at least 640 MS/s under the Nyquist criterion. High-order modulation also places strict requirements on converter noise and distortion. Pipelined ADCs are attractive for these applications. Their multistage architecture supports GS/s conversion while maintaining medium-to-high resolution [1,2,3,4,5].

The performance of a pipelined ADC is largely determined by the multiplying digital-to-analog converter (MDAC) employed in each pipeline stage. Within the MDAC, the residue amplifier (RA) must establish an accurate closed-loop gain within a limited settling interval. Finite amplifier gain gives rise to interstage gain error (IGE), whereas insufficient bandwidth and nonlinear settling introduce additional dynamic errors. These nonidealities distort the residue transfer characteristic and consequently degrade the signal-to-noise-and-distortion ratio (SNDR) and spurious-free dynamic range (SFDR).

The design challenge becomes increasingly pronounced in advanced CMOS technologies. As device dimensions shrink, the intrinsic gain of transistors generally decreases, making it more difficult to achieve both high gain and wide bandwidth under a reduced supply voltage. Process, voltage, and temperature (PVT) variations further perturb the amplifier gain and settling behavior. Although increasing the gain through analog design can alleviate IGE, it often incurs substantial power consumption and may restrict the achievable conversion speed. Digital background calibration has therefore become an important technique for maintaining the accuracy of high-speed pipelined ADCs without excessively increasing analog design complexity.

Various background IGE calibration techniques have been reported [6,7,8,9,10,11,12]. Reference-ADC [12], split-ADC [9], and summing-node-based approaches can achieve relatively rapid convergence [6], but they generally require additional analog circuitry, precise channel matching, or dedicated calibration paths. Their effectiveness may also deteriorate under particular input conditions. Off-chip optimization reduces the amount of on-chip digital hardware, but it cannot continuously track gain variations during normal operation [10]. Gagliardi et al. improved DAC static linearity by measuring unit-element mismatches and optimizing their selection, but this approach requires auxiliary mismatch sensing and is not directly applicable to continuous IGE tracking [13]. Li et al. combined genetic optimization with a neural network for joint-error calibration in a pipelined ADC. However, the method requires reference training data and relatively complex GA/NN computation [14]. By contrast, correlation-based background calibration injects a pseudorandom dither into the MDAC and estimates the gain error by correlating the dither sequence with the backend residue [8]. This approach requires only limited additional analog hardware and can operate concurrently with normal ADC conversion. Its convergence speed and steady-state accuracy, however, depend strongly on the adaptation algorithm.

Most correlation-based calibration schemes employ a fixed-step least mean squares (LMS) algorithm [15,16,17]. The adaptation step size directly determines the tradeoff between convergence speed and steady-state fluctuation. A large step size accelerates convergence but produces a relatively large estimation ripple after convergence. A small step size improves steady-state accuracy, although considerably more samples are required. Variable-step LMS algorithms can alleviate this tradeoff by dynamically adjusting the step size according to the estimation error. Sigmoid-based step-size functions are commonly adopted, but their exponential operations increase the complexity and power consumption of digital implementation. In addition, fluctuations generated by the coarse-estimation stage may propagate into the subsequent fine-estimation stage and limit the final accuracy. A practical calibration algorithm should therefore combine rapid convergence, stable refinement, and low implementation complexity.

This work presents a weight-regulated, dither-based hybrid dual-stage background calibration technique for a 1.5 GS/s 12-bit pipelined ADC. A 1-bit pseudorandom dither is injected into the first three MDAC stages, and the resulting correlation components are used to estimate the corresponding IGEs. The first calibration stage employs a large fixed-step LMS algorithm to obtain a rapid coarse estimate. The second stage further refines the estimate using a rational-quadratic adaptive-variable-step LMS algorithm, thereby avoiding the exponential operation required by conventional sigmoid-based functions. A weight-regulated error coefficient is introduced to suppress the influence of first-stage fluctuations on the final estimate.

## 2. Dither-Based Interstage Gain Error Calibration

### 2.1. Architecture of the 1.5 GS/s 12-bit Pipelined ADC

**Figure 1:**
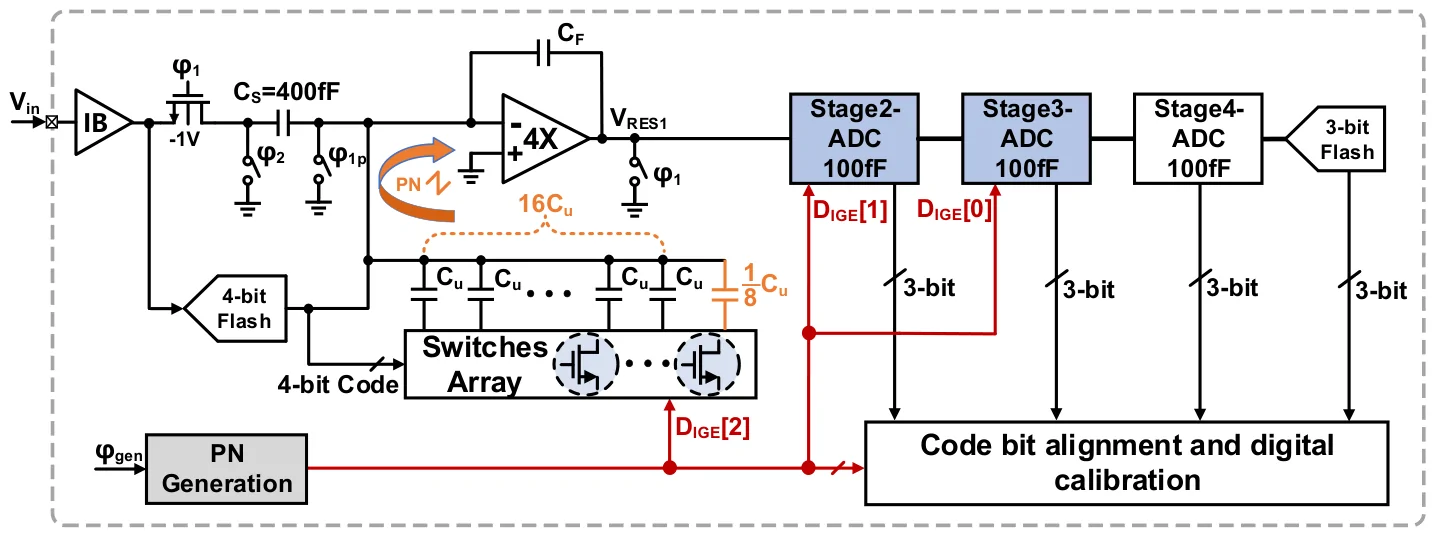
Architecture of the proposed 1.5-GS/s 12-bit pipelined ADC with dither-based interstage gain error calibration.

Figure 1 shows the architecture of the proposed 1.5 GS/s 12-bit pipelined ADC. Although a simplified single-ended signal path is shown for clarity, the implemented ADC operates differentially with a full-scale input range of 1.4 Vpp. The input buffer drives the 400 fF sampling capacitor C_S_, which samples the input signal during φ1. A 4-bit flash ADC generates the first-stage digital code and controls the capacitive digital-to-analog converter (DAC). The DAC output is subtracted from the sampled input, and the RA amplifies the resulting residue by four. The closed-loop gain is determined by the ratio of the sampling capacitor C_S_ and the feedback capacitor C_F_.

The first stage is followed by three 3-bit pipeline stages and a backend 3-bit flash ADC. Each subsequent pipeline stage has an input sampling capacitance of 100 fF. The digital outputs of all stages are delivered to the code-alignment and digital calibration block, where redundancy correction and IGE calibration are performed to generate the final 12-bit output code.

A pseudorandom sequence is generated on-chip and applied to the first-stage switches array. The corresponding dither-injection signals are denoted by D_IGE_<2>, D_IGE_<1>, and D_IGE_<0>, respectively. In each calibrated stage, the dither component is injected into the MDAC through a dedicated capacitor. The injected sequences are subsequently correlated with the backend digital output to estimate the corresponding IGEs. The resulting gain estimates are then used by the digital calibration block to correct the code weights of the first three stages.

Only the first three pipeline stages are calibrated because IGEs in the earlier stages have a stronger effect on the overall ADC performance. By contrast, the fourth-stage gain error has a limited effect on the output spectrum and its detection would also require a larger dither amplitude for fewer backend quantization levels remain.

**Figure 2:**
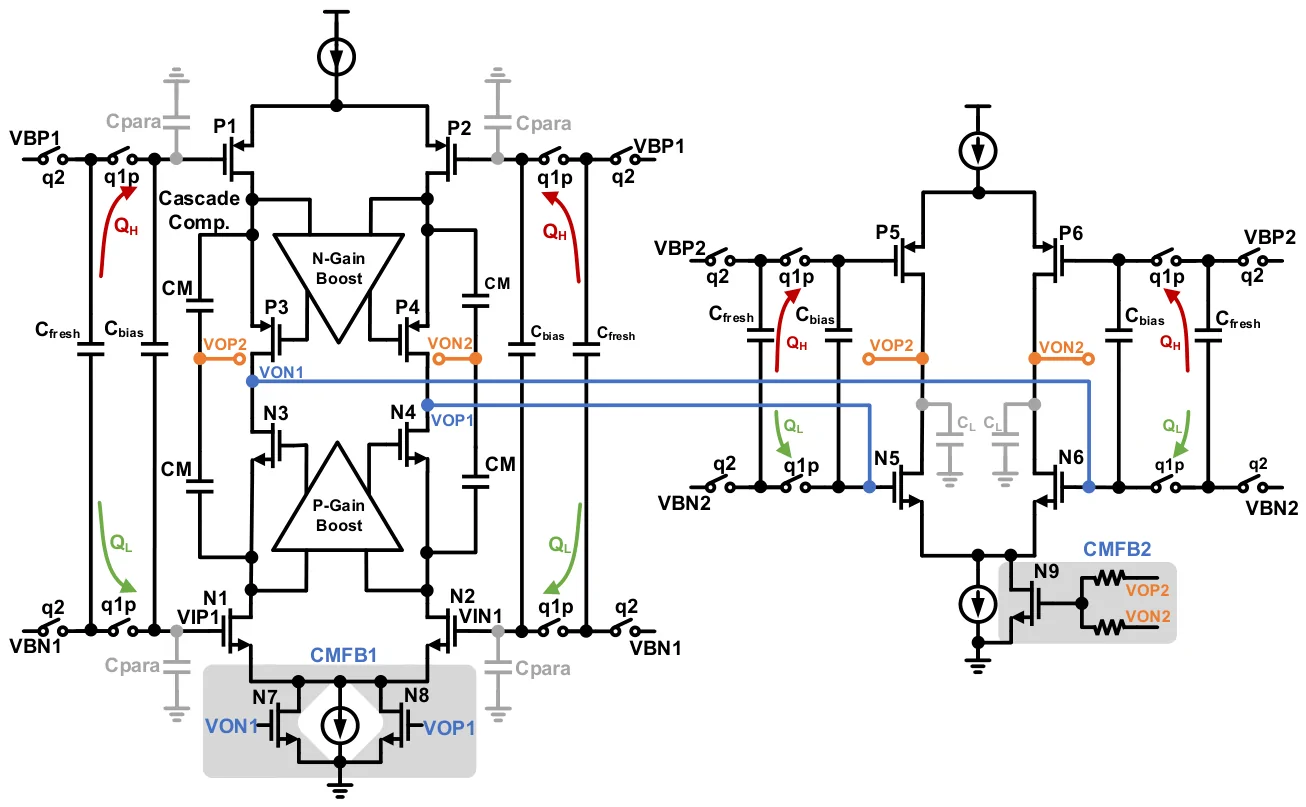
Transistor-level implementation of the residue amplifier.

The detailed RA implementation is shown in Figure 2. The first gain stage employs the differential input pair N1/N2 and P1/P2 together with cascode devices N3/N4 and P3/P4. The local N-type and P-type gain-boosting amplifiers regulate the cascode nodes and increase the effective output resistance, thereby improving the low-frequency gain. Switched-capacitor bias networks composed of C_fresh_, C_bias_, and the q1p/q2-controlled switches are used in both stages. The charge transfers Q_H_ and Q_L_ provide additional transient drive during residue amplification without relying solely on a larger static bias current. The second stage uses the complementary branches N5/N6 and P5/P6 in a push–pull configuration. This structure provides high open-loop DC gain and wide bandwidth in advanced CMOS technologies. The compensation capacitors C_M_ stabilize the two-stage amplifier, while CMFB1 and CMFB2 regulate the output common-mode voltages of the two stages.

**Figure 3:**
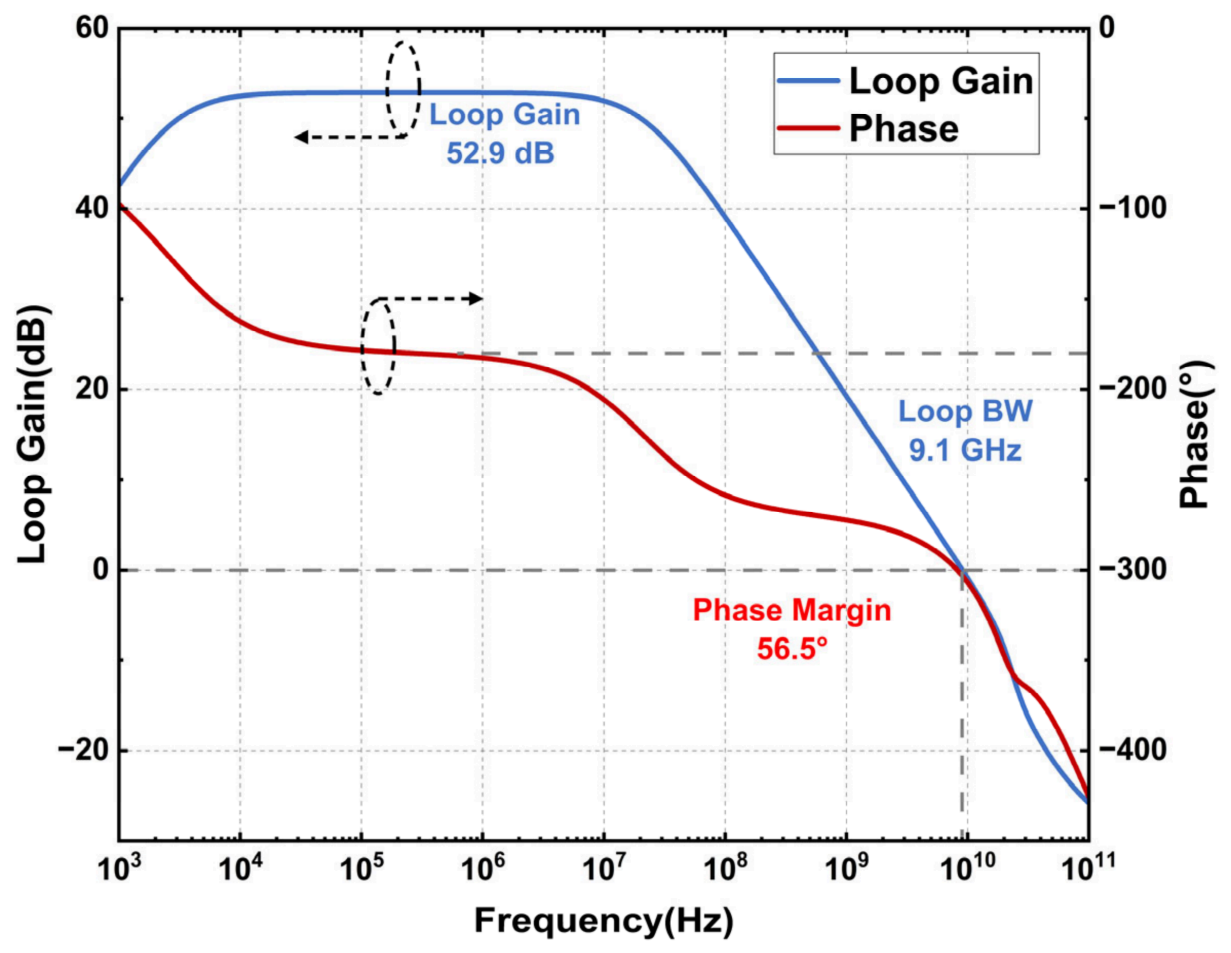
Simulated loop-gain magnitude and phase responses of the residue amplifier.

Figure 3 shows the simulated loop response of the RA. The low-frequency loop gain is 52.9 dB, and the loop bandwidth reaches 9.1 GHz. A phase margin of 56.5° is obtained, indicating stable closed-loop operation under the simulated condition. The wide loop bandwidth and push–pull output stage support rapid residue settling at the target sampling rate. However, a loop gain of 52.9 dB cannot ensure the required closed-loop gain accuracy for a 12-bit ADC over PVT variations. The remaining gain deviation manifests as an IGE and is subsequently quantized by the downstream pipeline stages, thereby introducing errors into the final digital output. Therefore, background IGE calibration is still required even with the gain-boosted RA [18].

**Figure 4:**
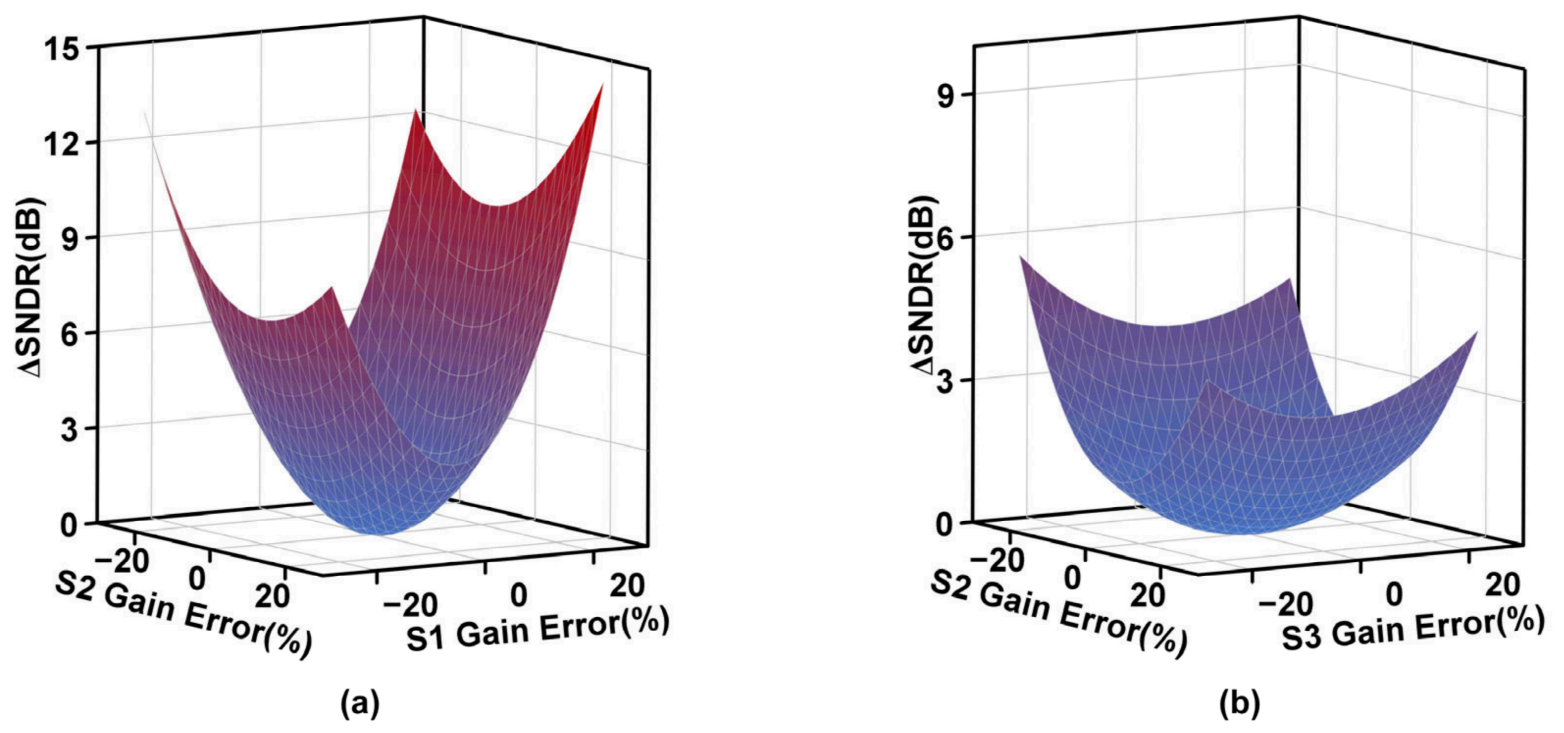
Simulated SNDR degradation as a function of the IGEs. (**a**) Gain errors in stages S1 and S2; (**b**) gain errors in stages S2 and S3.

Figure 4 illustrates the stage-dependent impact of interstage gain errors on the dynamic performance of the pipelined ADC, where S1, S2, and S3 denote the first three pipeline stages. In a pipelined architecture, errors originating from earlier stages carry larger output-referred weights, whereas errors introduced in later stages are progressively attenuated by the preceding interstage gains when referred to the ADC input. As shown in Figure 4a, the SNDR degradation increases rapidly as either the S1 or S2 gain error deviates from zero and reaches approximately 14 dB over the investigated range. The steeper variation along the S1 direction confirms the greater sensitivity to the first-stage gain error. By comparison, Figure 4b shows a maximum SNDR degradation of only approximately 5 dB for gain errors in S2 and S3.

**Figure 5:**
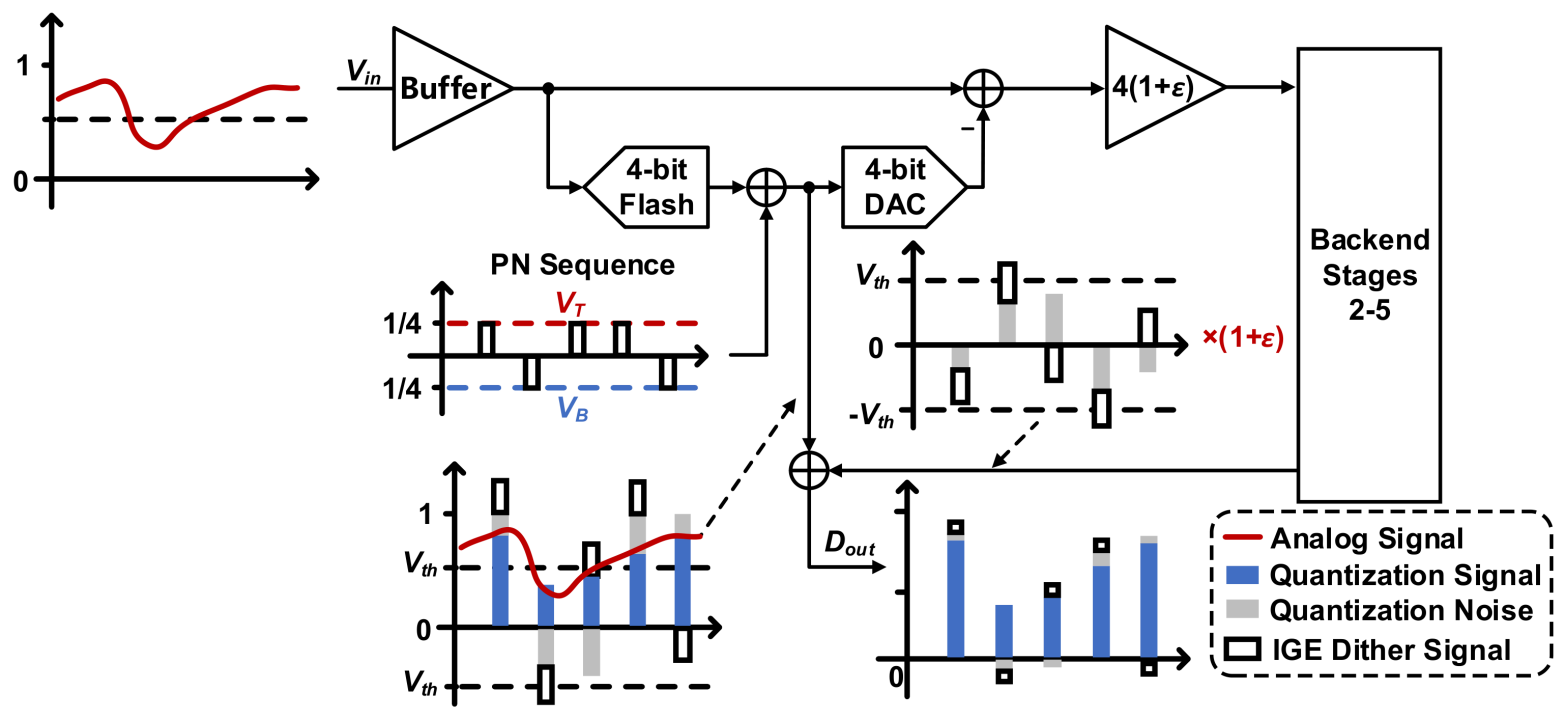
Performance deterioration due to IGE.

With the ideal interstage gain, the first-stage quantization error and the injected dither are cancelled during digital reconstruction. However, finite RA gain changes the nominal gain from 4 to 4(1 + ε), resulting in incomplete cancellation. A residual component related to the injected dither then propagates through the backend stages together with the residue quantization noise, as illustrated in Figure 5. Although this residual component degrades the SNDR and SFDR before calibration, it retains a statistical correlation with the known Pseudorandom Number (PN) sequence. By contrast, the input signal and backend quantization noise are uncorrelated with PN. Correlating the backend residue with PN therefore isolates the gain-dependent component and provides the basis for interstage gain estimation.

### 2.2. Interstage Gain Error Detection and Correction

**Figure 6:**
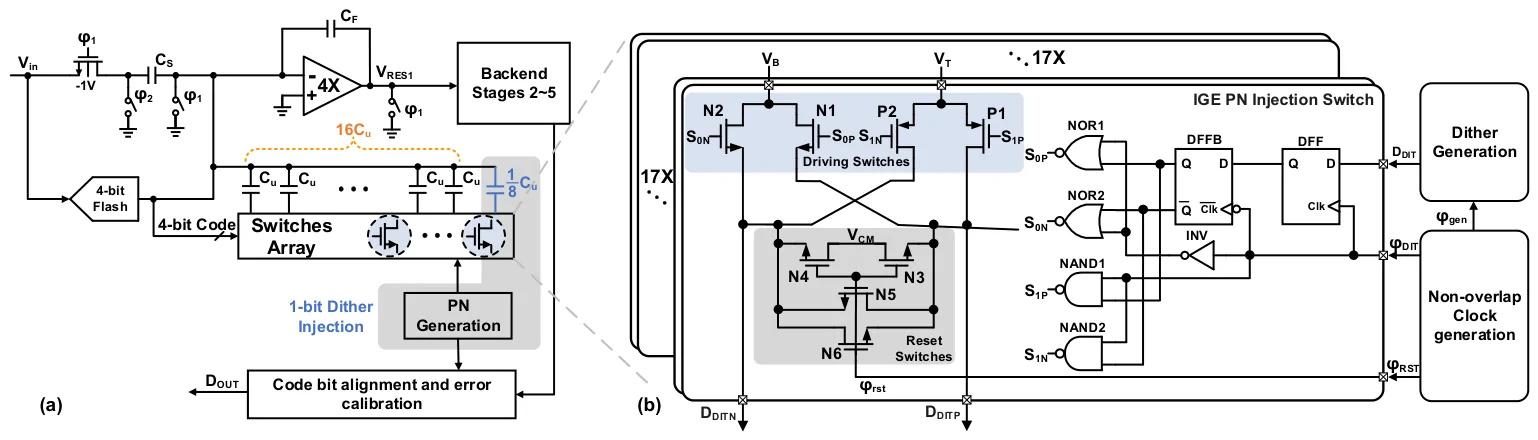
(**a**) Dither injection path in the first pipeline stage; (**b**) differential PN-controlled injection switches.

A small-amplitude 1-bit dither is injected into each of the first three pipeline stages. Figure 6a shows the injection path and its connection to the digital calibration block. In the first stage, the total DAC capacitance is 16×Cu, whereas the dedicated dither capacitor is Cu/8. The dither capacitance is therefore 1/128 of the total DAC capacitance. This ratio limits the disturbance applied to the residue while maintaining a detectable correlation component at the backend output. The PN sequence is generated on-chip and applied to the dither capacitors through the switch array.

Figure 6b illustrates the differential PN injection circuit used for IGE calibration. The PN sequence generated on-chip is synchronized with the MDAC operating phases by two cascaded D-type flip-flops. The retimed differential outputs are subsequently decoded by the NOR- and NAND-based logic network to generate the complementary control signals S_1P_, S_1N_, S_0P_, and S_0N_. This arrangement ensures that the PN state changes only within the designated switching interval and prevents asynchronous transitions from disturbing residue amplification.

The analog switching network consists of differential driving switches and a reset circuit. According to the PN polarity, the driving switches steer the two differential injection nodes toward V_T_ and V_B_ in opposite directions, thereby producing a bipolar dither component. During the reset phase, the reset switches pre-charge and equalize the two nodes to V_CM_. The driving and reset operations are controlled by nonoverlapping clocks, avoiding direct conduction between the reference voltages and suppressing switching transients. The resulting dither is injected through the dedicated MDAC capacitor during the residue-amplification phase and is synchronized with the main DAC switching sequence.

**Figure 7:**
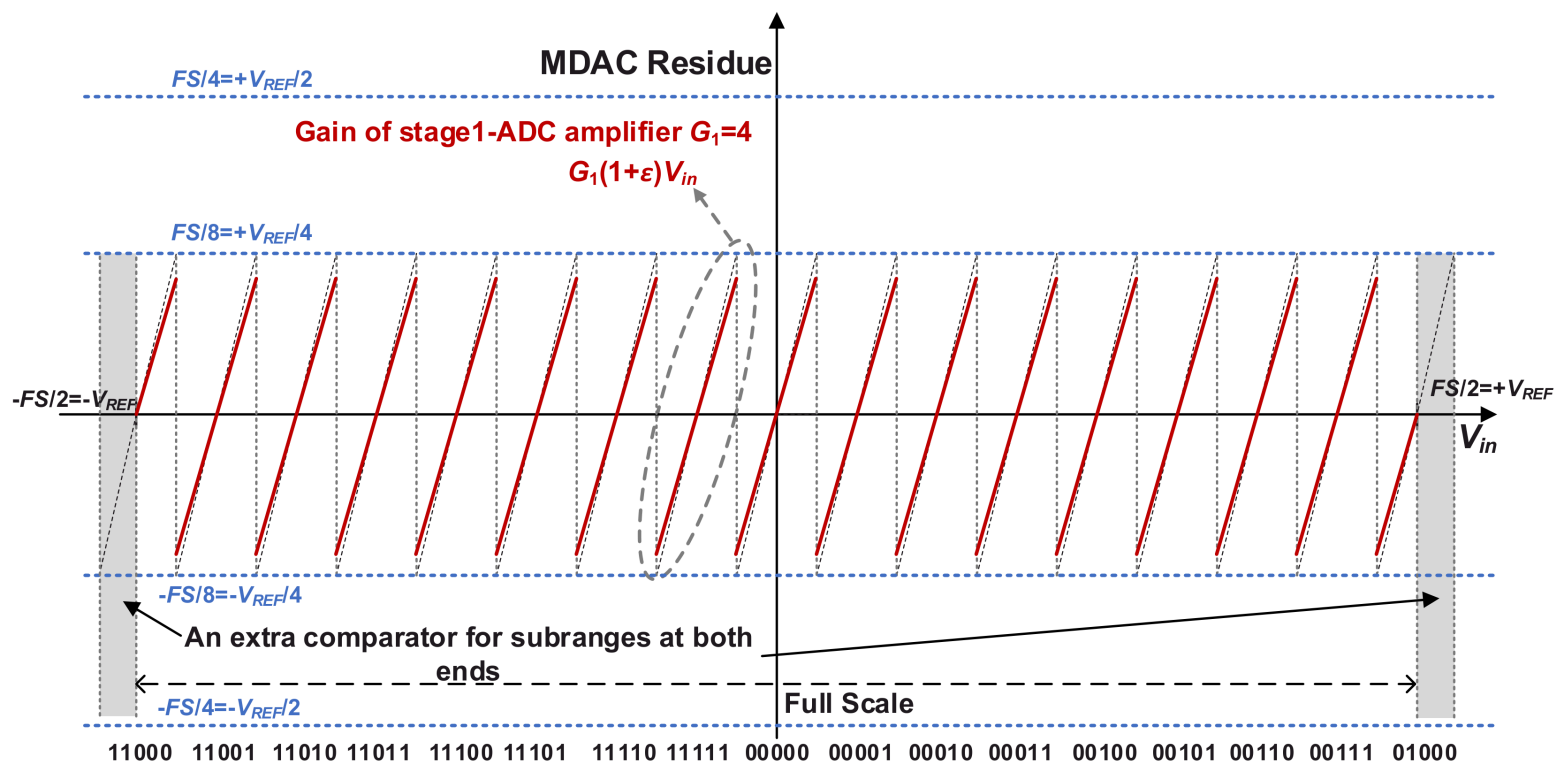
First-stage MDAC residue characteristic with additional comparators for identifying the boundary subranges during dither injection.

Dither injection must also be controlled near the limits of the input range. At either boundary, the injected perturbation may force the MDAC residue beyond the range accepted by the backend stages and consequently reduce the usable full-scale range. An additional comparator is inserted at both ends of the 4-bit sub-ADC input range to divide the two edge regions into 2 auxiliary subranges [19,20], as illustrated in Figure 7. This allows the dither to effectively perturb the injection stage while minimizing redundancy range consumption. Since the 1/128 dither amplitude only occupies 1/64 of the auxiliary subrange, it imposes no reduction on the usable full-scale input range. Meanwhile, the residue-transfer slope remains determined by the actual interstage gain, G_1_ = 4(1 + ε), so that the injected dither can still be used to extract the gain deviation while avoiding residue overload near the input boundaries.

For the first pipeline stage, the input signal superimposed with the dither is represented as *V_IN_*:

(1) $$V_{IN}=V_{in}+r\times PN$$

here *PN* denotes the pseudorandom number sequence, with values of 1 or −1, and *r* represents the analog amplitude injected into the MDAC. *D_res_*_1_ is the digital code for the first-stage residue *V_res_*_1_ quantized by the backend stages.

(2) $$D_{res1}=(1+\varepsilon )G_{i1}(V_{IN}-V_{DAC1})-Q_{res1}$$

*G_i_*_1_ is the ideal RA gain of stage 1, *ε* is the gain error, and *Q_res_*_1_ is the quantization error. By correlating *D_res_*_1_ with *PN*, the relationship can be formulated as follows:

(3) $$PN\times D_{res1}=r\times (1+\varepsilon )G_{i1}\times PN^{2}-PN\times Q_{res1}+PN\times (1+\varepsilon )G_{i1}\times (V_{in}-V_{DAC1})$$

Because PN is uncorrelated with the input signal and the backend quantization error, the last two terms in Equation (3) approach zero after correlation averaging. Moreover, E(PN^2^) = 1, so the actual interstage gain can therefore be estimated as

(4) $${\hat{G}}_{err1}=(1+\varepsilon )G_{i1}=\frac{E(PN\times D_{res1})}{r}$$

Once the actual gain ${\hat{G}}_{err1}$ is estimated, the calibration is performed for residue code *D_res_*_1_ as follows:

(5) $$D_{res1\_cal}=\frac{G_{i1}}{{\hat{G}}_{err1}}D_{res1}$$

*D_res_*_1*_cal*_ is the calibrated *D_res_*_1_. The second and third stages use the same estimation and correction procedure. Calibration proceeds from the backend toward the input. Thus, the third stage is calibrated first, followed by the second and first stages. This order prevents uncorrected backend gain errors from biasing the estimates of the preceding stages.

## 3. Proposed Hybrid Dual-Stage Algorithm

The gain obtained from the dither correlation in Equation (4) contains random fluctuations caused by the input signal, quantization noise, and the finite correlation window. An LMS loop can suppress these fluctuations by iteratively updating the gain estimate. However, its performance is strongly dependent on the selected step size.

**Figure 8:**
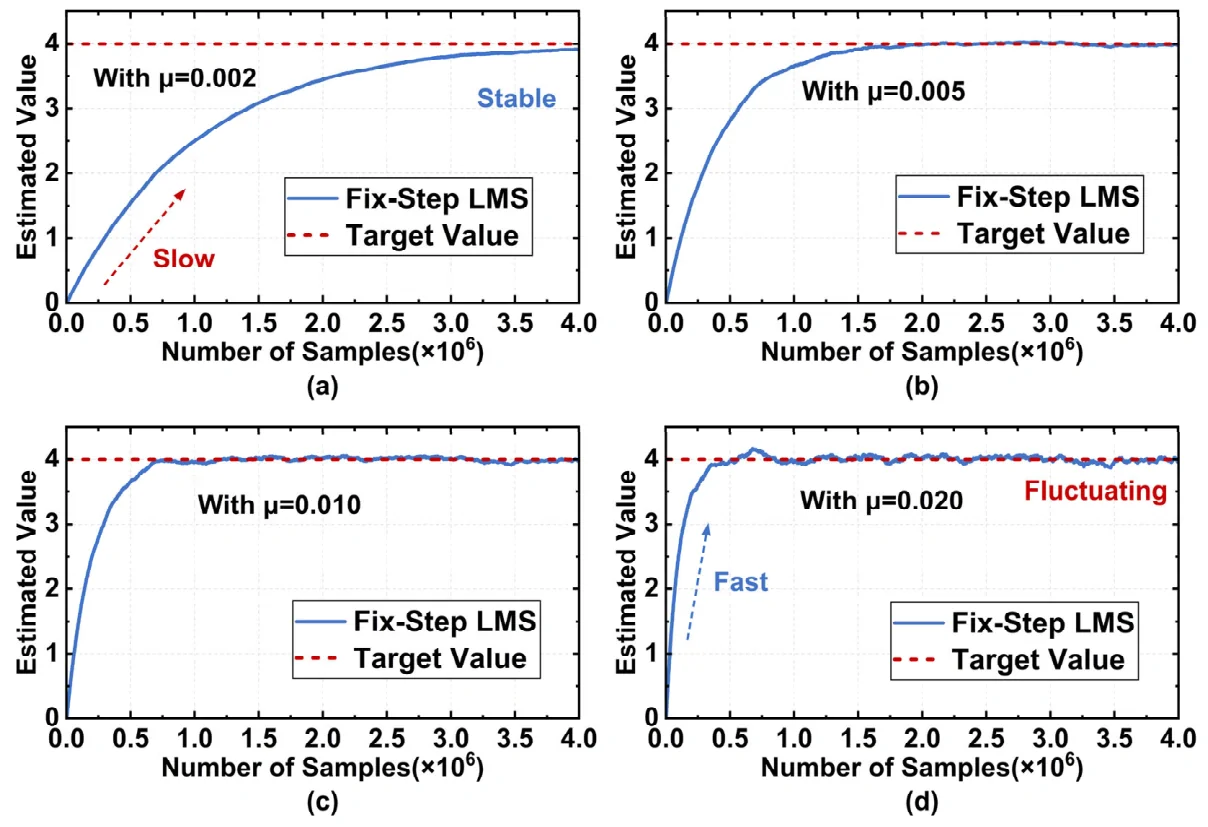
Convergence behavior of the conventional fixed-step LMS algorithm for: (**a**) μ = 0.002; (**b**) μ = 0.005; (**c**) μ = 0.010; (**d**) μ = 0.020.

The convergence behavior of a conventional fixed-step LMS algorithm is compared in Figure 8. A small step size, such as μ = 0.002, produces a smooth estimate but requires a large number of samples to approach the target value. Increasing the step size accelerates convergence, as observed for μ = 0.005 and 0.010. However, an excessively large value, such as μ = 0.020, causes overshoot and considerable steady-state fluctuation. A single fixed step size therefore cannot simultaneously provide rapid convergence and a stable final estimate.

**Figure 9:**
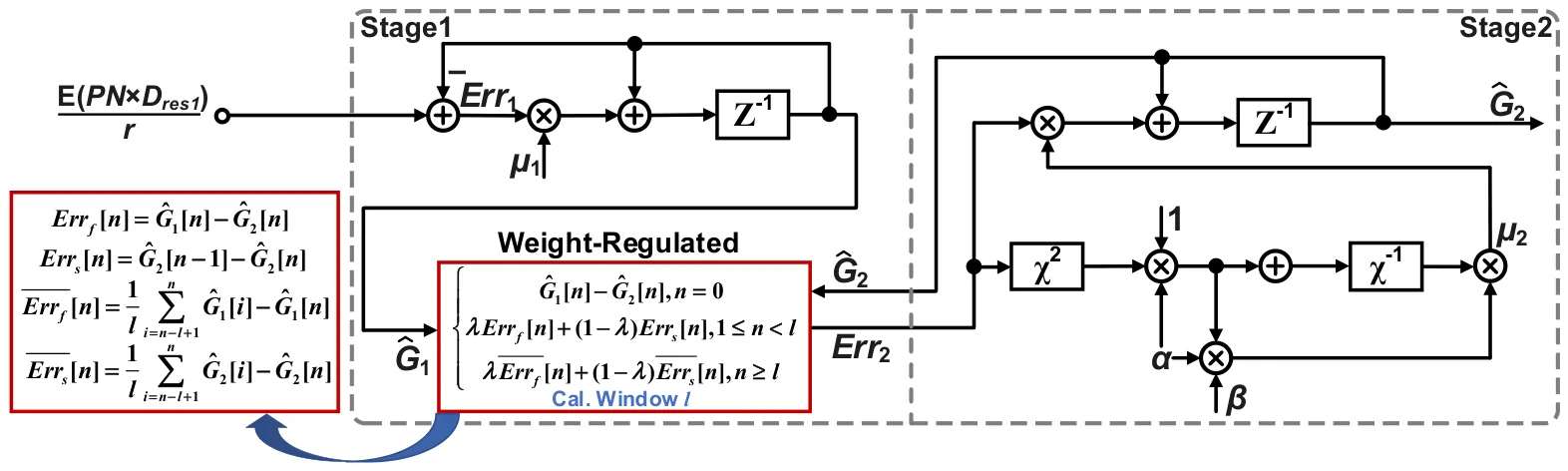
Block diagram of the proposed weight-regulated hybrid dual-stage calibration algorithm.

To alleviate this trade-off, the proposed calibration adopts the weight-regulated hybrid dual-stage structure shown in Figure 9. A large fixed-step LMS loop provides rapid coarse estimation, while a rational-quadratic adaptive-variable-step (RQ-AVS) LMS loop refines the result. The weighting coefficient λ suppresses the influence of first-stage fluctuations while preserving rapid overall convergence. Both stages operate continuously to achieve fast convergence and a stable final estimate.

In the first stage, a fixed-step LMS algorithm is employed to rapidly estimate the gain error. The error coefficient and the iterative estimation formula are expressed as follows:

(6) $$Err_{1}[n]=\frac{E(PN\times D_{res1})}{r}-{\hat{G}}_{1}[n]$$

(7) $${\hat{G}}_{1}[n+1]={\hat{G}}_{1}[n]+\mu_{1}\times Err_{1}[n]$$

The second stage employs an adaptive-variable-step (AVS) LMS algorithm to stabilize and filter the results from the first stage. Conventional AVS LMS algorithms are frequently based on the Sigmoid function, as expressed in Equation (8), where β controls the maximum step size, and α determines the adaptation rate. However, the exponential operation involving the natural base e in the Sigmoid function poses significant challenges and high costs for implementation in digital integrated circuits. In this paper, the RQ-AVS method is proposed, as shown in Equation (9):

(8) $$\mu_{2}[n]=\beta (\frac{1}{1+e^{-\alpha \left|Err_{2}[n]\right|}}-\frac{1}{2})$$

(9) $$\mu_{2}[n]=\frac{\alpha \times \beta \times {Err_{2}}^{2}[n]}{1+\alpha \times {Err_{2}}^{2}[n]}$$

The proposed function depends on the squared error and approaches β as the error magnitude increases. When the error approaches zero, μ_2_[n] also approaches zero. The second-stage update can consequently provide rapid tracking during the initial calibration and small corrections near convergence. Compared with the sigmoid function, Equation (9) replaces the exponential calculation with rational-quadratic operations, making it more suitable for digital hardware implementation.

**Figure 10:**
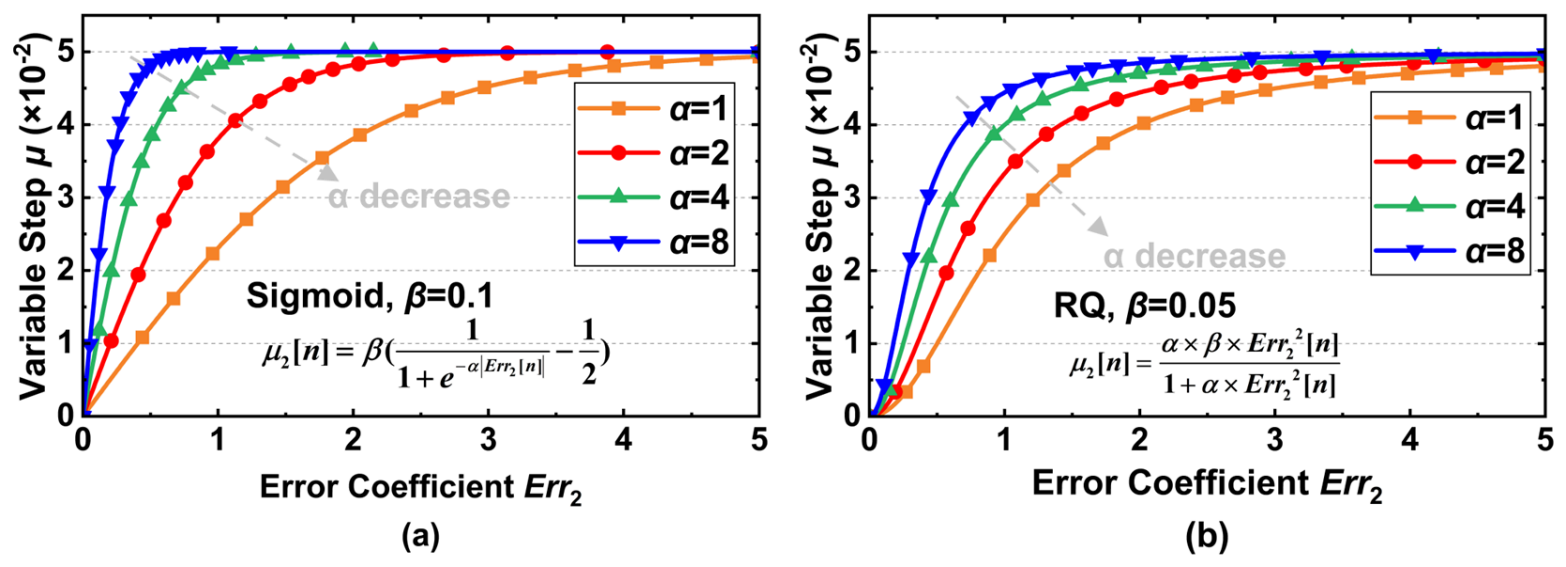
Variable step μ curve of stage-2. (**a**) Based on sigmoid function; (**b**) based on the proposed rational-quadratic form.

Figure 10 compares the step-size characteristics of the sigmoid and RQ-AVS functions. Both functions provide a monotonic transition from a small step near zero error to a saturated step at large error. The parameter α controls the transition rate. A larger α produces a faster increase, whereas a smaller value provides a smoother transition. The proposed RQ function therefore retains the required adaptive behavior while avoiding the exponential term.

If the second-stage error is defined only as the difference between the coarse and fine estimates, fluctuations in the first-stage output are directly transferred to the second-stage adaptation. A weight-regulated error function is introduced to reduce this dependence. For compactness, G_1_[n] and G_2_[n] denote the first- and second-stage gain estimates in the following equations. The second-stage error is defined as

(10) $$\begin{array}{cc} Err_{2}[n] & =g(G_{1}[n],G_{2}[n])\\ & =\left\{\begin{array}{c} G_{1}[n]-G_{2}[n],n=0\\ \lambda Err_{f}[n]+(1-\lambda )Err_{s}[n],1\leq n<l\\ \lambda \bar{Err_{f}}[n]+(1-\lambda )\bar{Err_{s}}[n],n\geq l \end{array}\right. \end{array}$$

(11) $$Err_{f}[n]=G_{1}[n]-G_{2}[n]$$

(12) $$Err_{s}[n]=G_{2}[n-1]-G_{2}[n]$$

(13) $$\bar{Err_{f}}[n]=\frac{1}{l}\sum_{i=n-l+1}^{n}G_{1}[i]-G_{1}[n],n\geq l$$

(14) $$\bar{Err_{s}}[n]=\frac{1}{l}\sum_{i=n-l+1}^{n}G_{2}[i]-G_{2}[n],n\geq l$$

The error parameter is regulated by *λ* in [0, 1], which weights the contributions of first- and second-stage deviations. *l*, defined as the moving average window, aims to suppress instantaneous jitters. A larger *λ* increases the weights of Equations (11) and (13), while decreasing the weights of Equations (12) and (14). A proper *λ* effectively decouples the fine estimation from first-stage fluctuations, thereby improving steady-state accuracy.

To quantitatively evaluate the digital hardware complexity, Table 1 compares the hardware resource overhead of conventional Sigmoid approximations against the proposed RQ-AVS method. While Sigmoid functions rely heavily on memory-intensive LUTs or multiplier-heavy polynomial approximations, the proposed RQ function is natively hardware-friendly. In our digital implementation, when α and β are chosen to be in the form of 2^n^ or 2^(−n)^, constant multiplications can be implemented via bit-shifting operations, eliminating the need for additional constant multipliers. This optimized RQ architecture replaces general multiplications with simple bit-shifting, reducing the core computational requirement to just one multiplier, one divider, two shift logics, and one adder. This structural simplification successfully bypasses the exponential overhead, resulting in low dynamic power consumption and rendering it highly suitable for purely on-chip background calibration.

Adaptive LMS algorithms generally encompass multi-step (discrete) and variable-step (continuous) methods. The proposed hybrid structure advances beyond them in three key aspects. First, unlike single-stage variable-step algorithms, the proposed dual-stage approach uses a fixed-step coarse stage to significantly accelerate overall convergence, with only a marginal trade-off in digital area. Second, conventional continuous variable-step methods rely on Sigmoid functions, which require resource-intensive digital approximations (e.g., LUTs, piecewise polynomials, or CORDIC algorithms). The proposed RQ-AVS avoids this overhead by utilizing highly efficient rational-quadratic operations. Finally, the weight-regulated parameter λ introduces an independent degree of freedom. By optimizing λ, the system effectively decouples the coarse-stage noise from the fine-estimation path without compromising the overall convergence speed.

## 4. Measurement Results and Discussion

**Figure 11:**
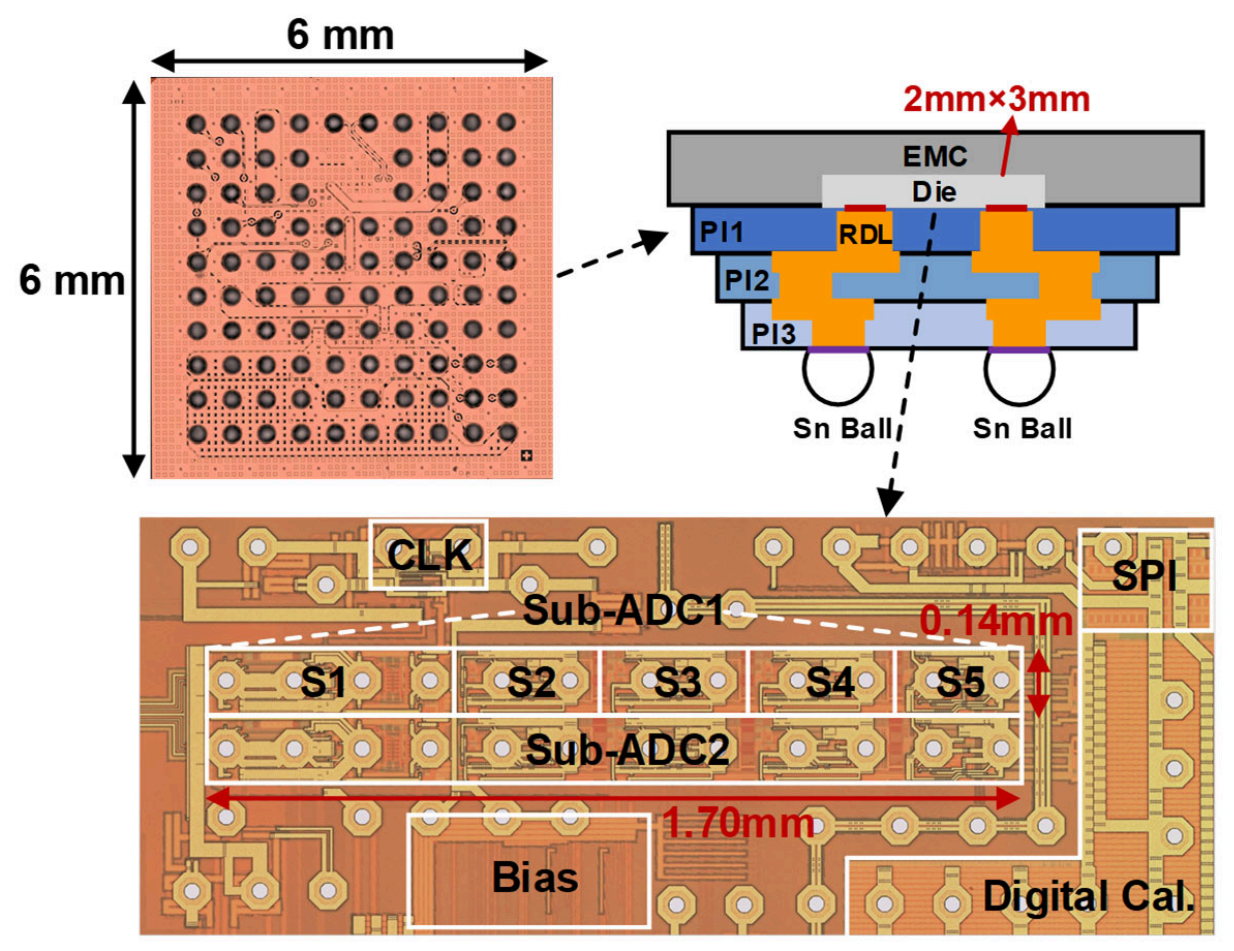
Die micrograph and fan-out wafer-level package of the fabricated ADC.

To experimentally validate the proposed background IGE calibration technique, a prototype 12-bit pipelined ADC was fabricated in a 28-nm CMOS process and measured at a sampling rate of 1.5 GS/s. The ADC employs a fully differential signal path with a 1.4-Vpp full-scale input range. As shown in Figure 11, the active single-channel ADC core occupies 1.70 mm × 0.14 mm, corresponding to an area of approximately 0.24 mm^2^. The die was assembled in a 6 mm × 6 mm flip-chip fan-out wafer-level package comprising three redistribution layers and three polyimide layers.

**Figure 12:**
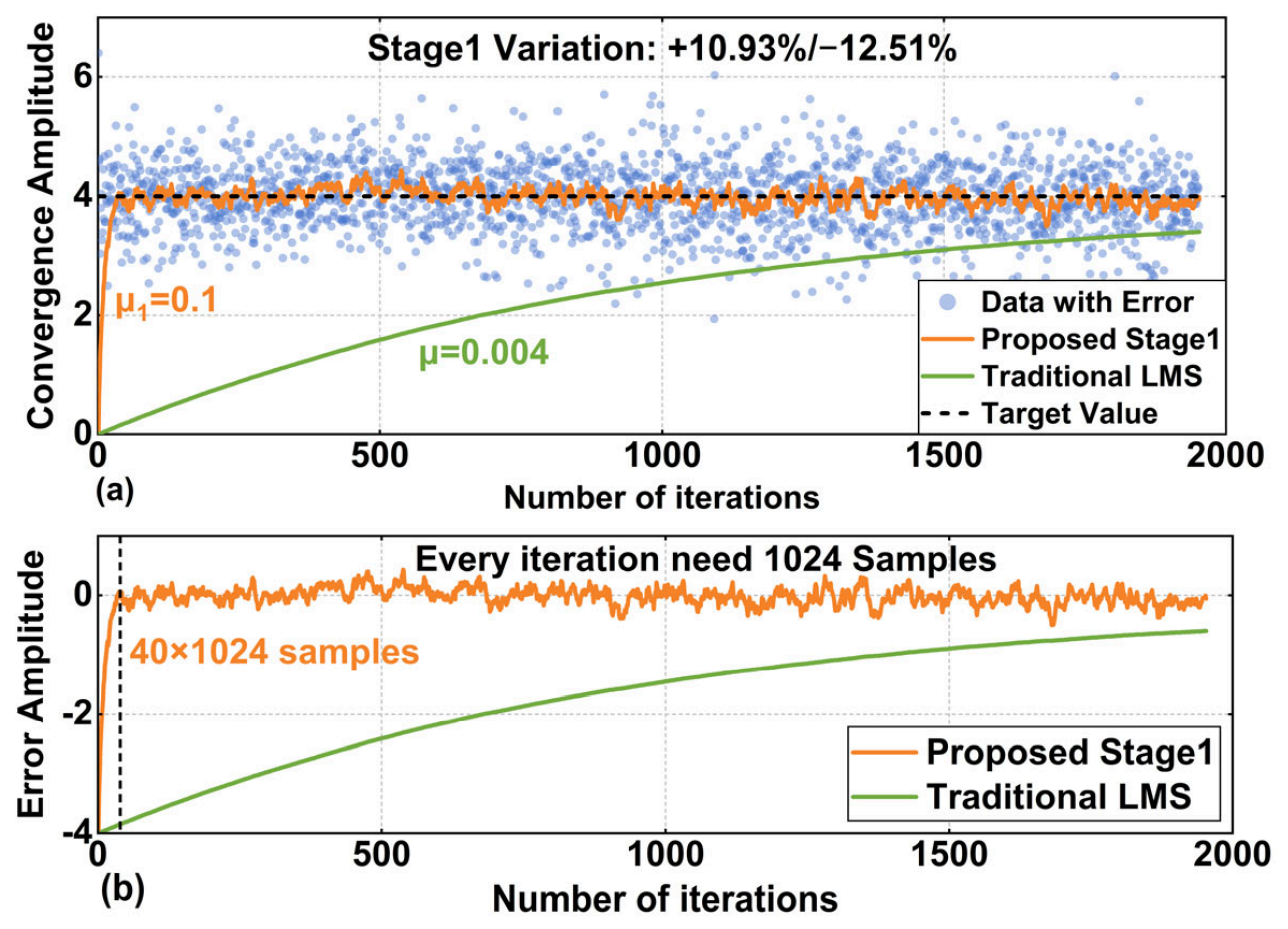
First-stage calibration convergence: (**a**) gain estimate; (**b**) estimation error.

**Figure 13:**
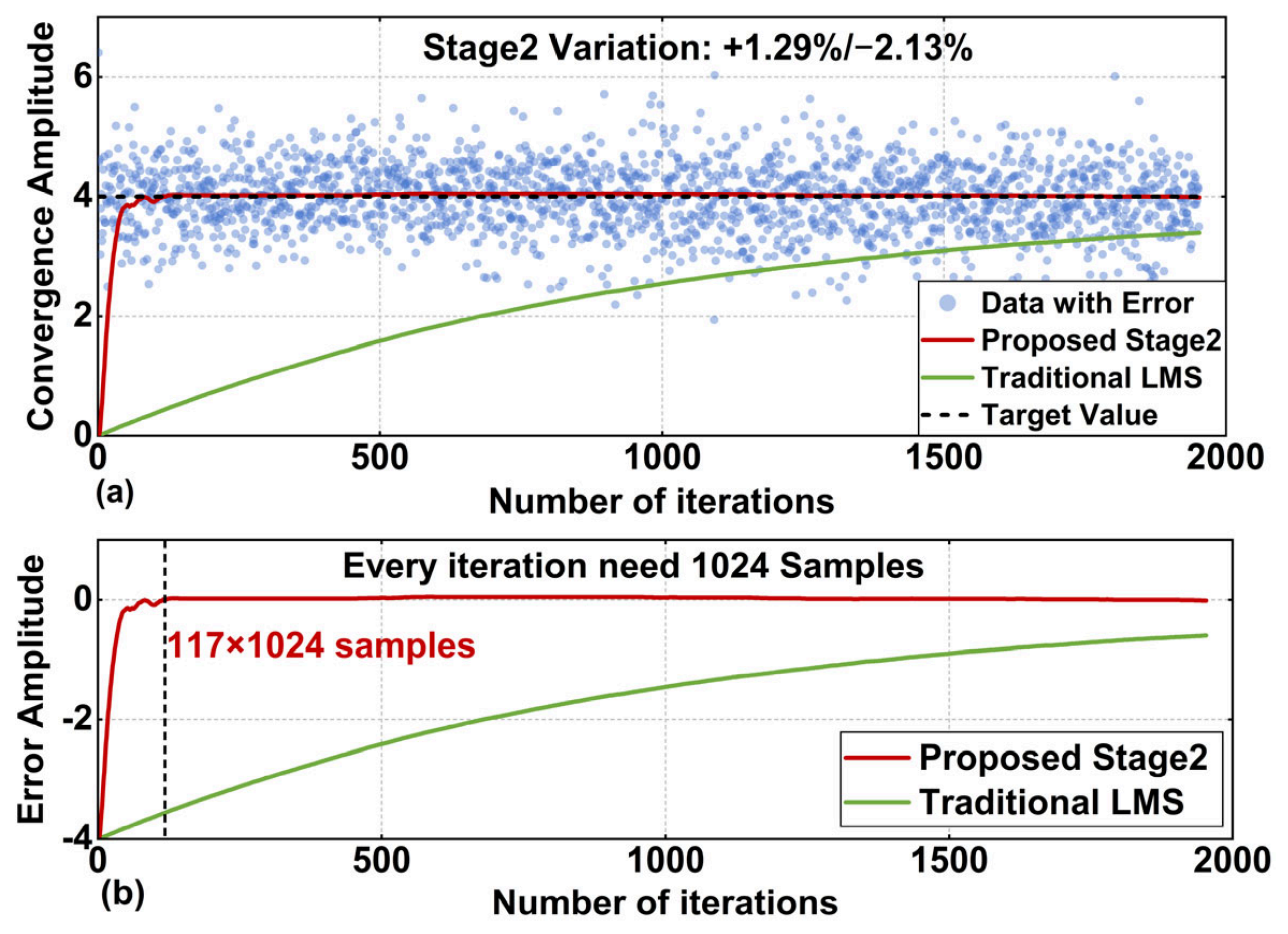
Second-stage calibration convergence: (**a**) gain estimate; (**b**) estimation error.

Figure 12 and Figure 13 illustrate the convergence results of the proposed hybrid dual-stage algorithm. As shown in Figure 12, the first stage uses a large step size to achieve rapid initial convergence within about 41k samples, but exhibits large steady-state fluctuations of +10.93%/−12.51%. Following stabilization and filtering in the second stage, as shown in Figure 13, the overall calibration fully converges at 120k samples, stabilizing the steady-state ripple at +1.29%/−2.13%.

**Figure 14:**
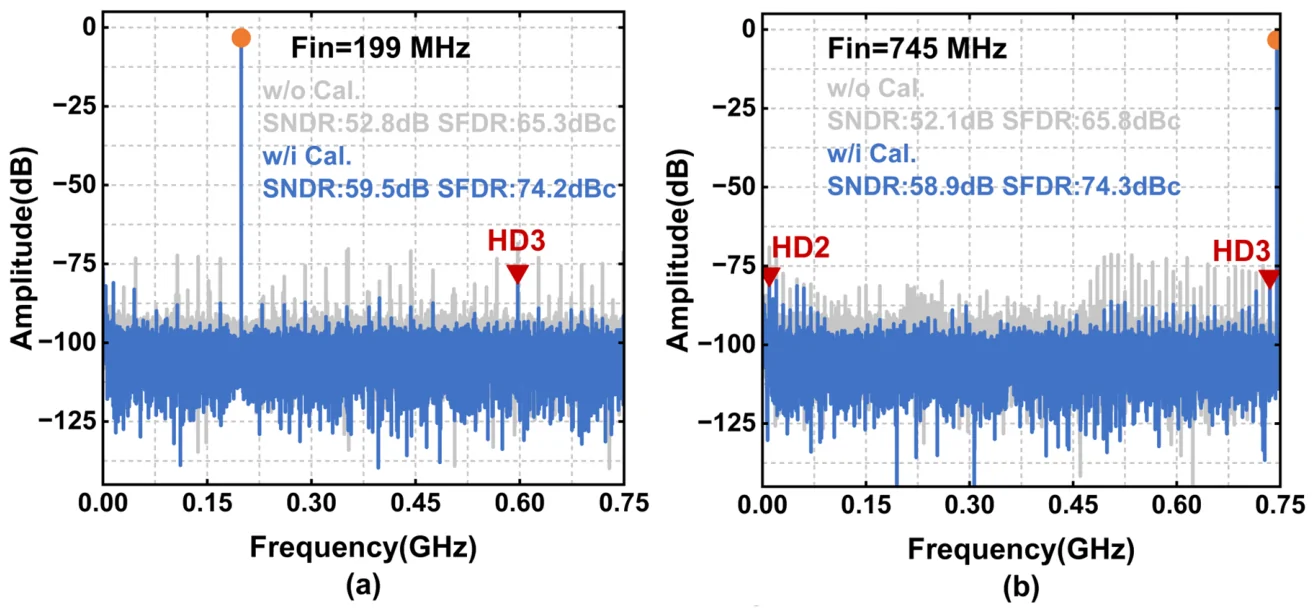
Measured output spectra. (**a**) At 199 MHz input; (**b**) at 745 MHz input.

Figure 14a and Figure 14b show the measured output spectra at input frequencies of 199 MHz and 745 MHz, respectively. At 199 MHz, enabling the proposed calibration improves the SNDR from 52.8 dB to 59.5 dB. The SFDR increases from 65.3 dBc to 74.2 dBc. The improvement is primarily attributed to the effective suppression of the IGE-induced noise-floor elevation and spurious components.

A similar performance improvement is observed for an input near the Nyquist frequency. The ADC output codes were stored in the on-chip Static Random-Access Memory (SRAM) and subsequently read through Serial Peripheral Interface (SPI). The captured data were processed offline using an 18,000-point FFT with a rectangular window, corresponding to a frequency-bin spacing of approximately 83.3 kHz at a sampling rate of 1.5 GS/s. At 745 MHz, the measured SNDR increases from 52.1 dB to 58.9 dB, while the SFDR improves from 65.8 dBc to 74.3 dBc. Before calibration, the spectrum exhibits an elevated noise floor and pronounced spurious components caused by IGEs. These spectral impairments are effectively suppressed after calibration, demonstrating accurate correction of the IGE-induced residue distortion. Moreover, the calibrated SNDR decreases only slightly, from 59.5 dB at 199 MHz to 58.9 dB at 745 MHz, indicating that the proposed calibration maintains its effectiveness as the input frequency approaches the Nyquist limit.

**Figure 15:**
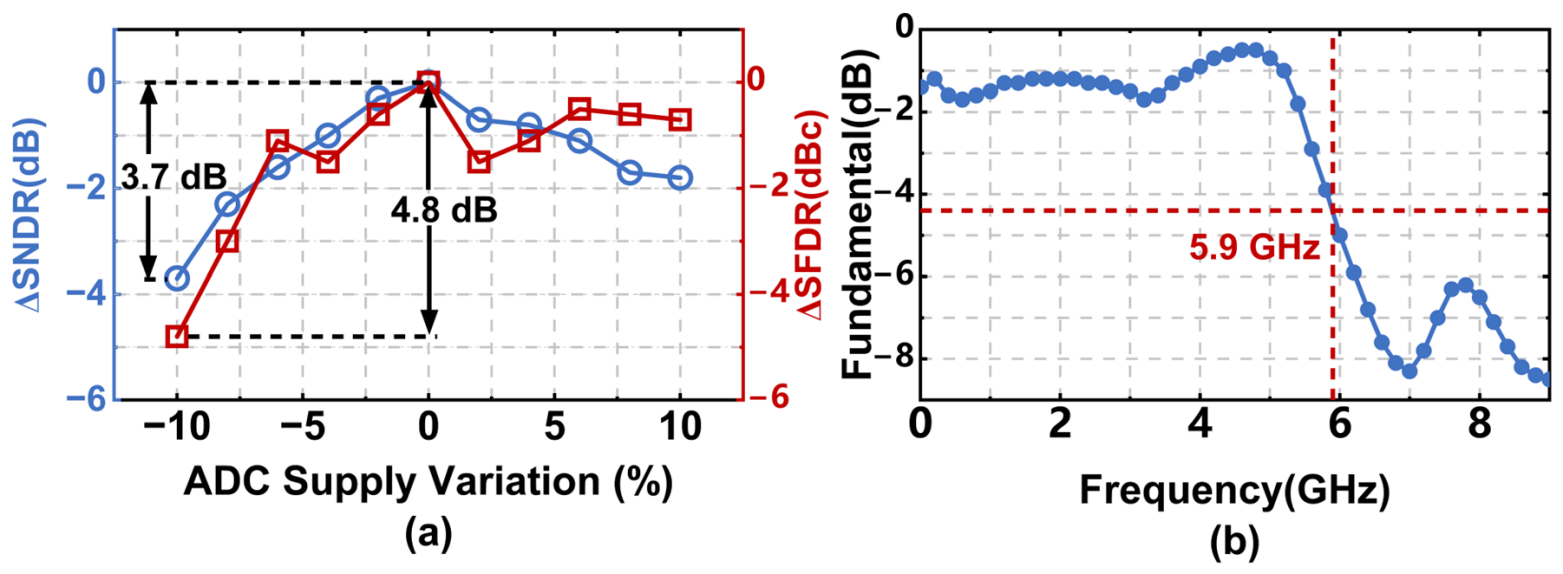
(**a**) Supply-voltage sensitivity; (**b**) bandwidth.

Figure 15a presents the measured sensitivity to ADC supply variation. The results are normalized to the performance at the nominal core supply voltage 1 V. Over a supply variation from −10% to +10%, the maximum SNDR and SFDR changes are 3.7 dB and 4.8 dB, respectively. Most of the degradation occurs at the lowest supply voltage, where the available amplifier headroom and settling capability are reduced. For positive supply variations, both metrics remain closer to their nominal values. These results show that the calibration can tolerate moderate supply variation, although the analog signal path remains sensitive to a substantial supply reduction.

The measured bandwidth is shown in Figure 15b. The ADC maintains a relatively flat fundamental amplitude over the first Nyquist band and continues to sample inputs well beyond the 1.5 GS/s sampling rate. Based on the criterion of −3 dB, the measured input bandwidth is approximately 5.9 GHz. This bandwidth allows the prototype to process high-frequency input signals and supports operation in higher Nyquist zones.

**Figure 16:**
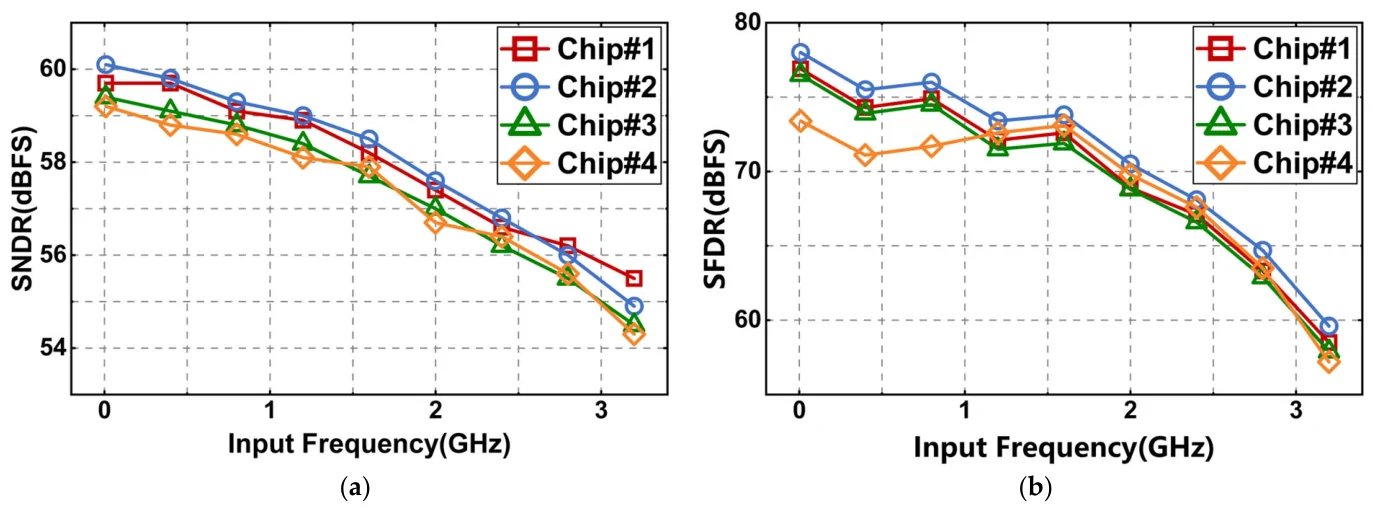
Chip-to-chip dynamic performance: (**a**) SNDR; (**b**) SFDR.

The chip-to-chip dynamic performance is evaluated using four packaged samples. As shown in Figure 16, the measured SNDR and SFDR are approximately 59−60 dB and 75−78 dBc at low input frequencies, respectively, and gradually degrade at higher frequencies due to sampling errors, input-network loss, and incomplete settling. The close agreement among the four samples demonstrates good chip-to-chip consistency. This consistency also indicates that the proposed background calibration can effectively track and compensate for interstage variations caused by random mismatch.

**Figure 17:**
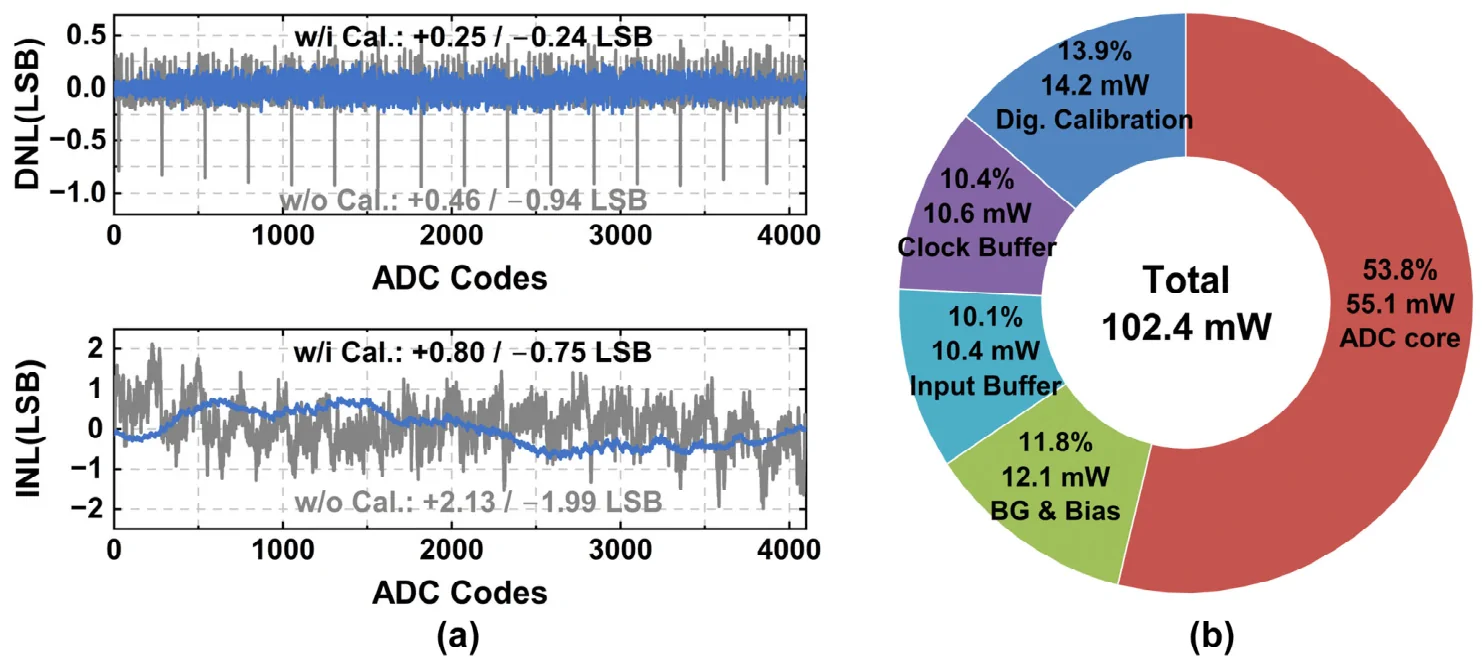
Measured linearity and power consumption: (**a**) DNL and INL with/without calibration; (**b**) power breakdown.

Figure 17a compares the measured static linearity before and after the proposed calibration. Before calibration, the uncorrected interstage gain errors lead to a differential nonlinearity (DNL) of +0.46/−0.94 LSB and an integral nonlinearity (INL) of +2.13/−1.99 LSB. After enabling the background calibration, the DNL significantly improves to a range of +0.25 to −0.24 LSB with no missing codes observed, and the INL is reduced to a range of +0.80 to −0.75 LSB. These comparative results indicate that the residue-gain correction effectively restores the code-width linearity required for 12-bit conversion.

Figure 17b summarizes the measured power consumption. The ADC consumes 102.4 mW in total. The ADC core consumes 55.1 mW, accounting for 53.8% of the total power. The digital calibration consumes 14.2 mW, or 13.9%. The bandgap and bias circuits consume 12.1 mW, while the input and clock buffers consume 10.4 mW and 10.6 mW, respectively. The calibration therefore introduces a measurable power overhead, but it enables an SNDR improvement of 6.8 dB at the near-Nyquist input.

**Figure 18:**
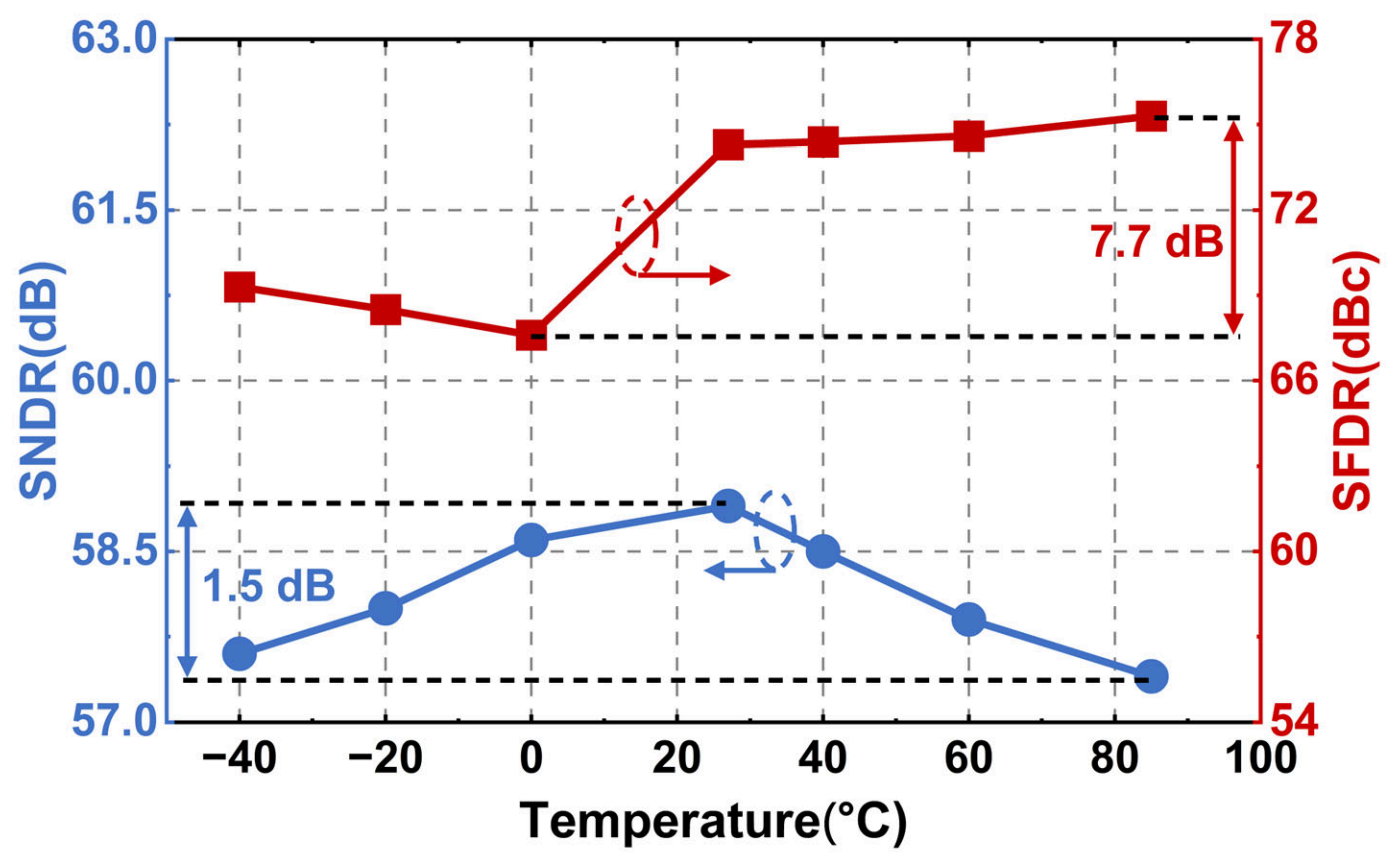
Estimated SNDR and SFDR versus temperature at a sampling rate of 1.5 GS/s and an input frequency of 745 MHz.

To evaluate the temperature robustness, the ADC prototype was tested from −40 °C to 85 °C. As shown in Figure 18, the measured SNDR and SFDR vary by a maximum of only 1.5 dB and 7.7 dB, respectively, across the entire 125 °C span. These results demonstrate that the calibrated ADC exhibits low sensitivity to temperature variations and maintains robust dynamic performance.

Table 2 compares the prototype with recently reported Nyquist-rate ADCs. At the near-Nyquist input, this work achieves an SNDR of 58.9 dB, corresponding to an effective number of bits of 9.49. The measured SFDR is 74.3 dBc. Among the selected works, the proposed ADC provides the highest near-Nyquist SNDR and a competitive SFDR while supporting on-chip background amplifier calibration. The Schreier figure of merit (FoM_S_) is 157.6 dB, and the Walden figure of merit (FoM_W_) is 94.8 fJ/conversion-step.

## 5. Conclusions

This paper presents a weight-regulated, dither-based hybrid dual-stage background calibration technique for IGEs in a 1.5 GS/s 12-bit pipelined ADC. A 1-bit pseudorandom dither is injected into the first three pipeline stages, and the gain errors are extracted through correlation with the backend residue. The first calibration stage employs a large fixed-step LMS algorithm to provide rapid coarse estimation, while the second stage uses an RQ-AVS LMS algorithm to refine and stabilize the result. A weight-regulated error function further suppresses fluctuations transferred from the first stage with only a minor penalty in convergence speed. The fabricated prototype ADC achieves an SNDR of 58.9 dB and an SFDR of 74.3 dBc at a 745 MHz input. The calibration converges within approximately 0.12 million samples and reduces the steady-state gain-estimation variation from +10.93%/−12.51% to +1.29%/−2.13%. The measured results demonstrate that the proposed technique provides fast and stable background IGE calibration for high-speed pipelined ADCs.

## Figures and Tables

**Table 1:** Comparison of Hardware Resource Overhead for Different Variable Step-Size Methods.

Method	Main Operations	Computational Resources	Storage Resources	Power Overhead
**Sigmoid LUT**	Pre-stored $\mu_{2}(err_{2})$	Address decoding, table-lookup logic	~16.4 kb ROM	Medium
**Sigmoid Piecewise Linear Approx.**	Interval judgment, slope multiplication, bias addition	~8 comparators, 1 multiplier, 2 adders	~256 bit ROM	Medium
**Sigmoid Polynomial Approx.**	$a_{1}err_{2}+a_{2}{err_{2}}^{2}+a_{3}{err_{2}}^{3}$	3~4 multipliers, 3~4 adders	A few coefficient registers	High
**RQ-AVS**	$\mu_{2}[n]=\frac{\beta \cdot \alpha \cdot {err_{2}}^{2}[n]}{1+\alpha \cdot {err_{2}}^{2}[n]}$	3 multipliers, 1 divider, 1 adder	A few coefficient registers	Medium
Optimized RQ-AVS *	Squaring, bit-shifting, addition, division	1 multiplier, 1 divider, 2 shift logics, 1 adder	A few coefficient registers	Low

*: When α and β are set to 2^n^ or 2^(−n)^, constant multiplications can be realized through bit-shifting, requiring no extra hardware multipliers.

**Table 2:** Performance summary and comparison.

References		This Work	M. Gu [21] ISSCC′25	Q. Yu [22] A-SSCC′25	L. Ricci [23] VLSI′23	A. Ali [24] ISSCC′20
Architecture		**Pipe.**	Pipe.	4×TI Pipe-SAR	8×TI SAR	4×TI Pipe.
Process (nm)		**28**	28	28	28	14
Supply (V)		**1**	1	0.9/2.5	1/1.4	1
ADC Core Area (mm^2^)		**0.24**	0.04	0.1	0.54	7.2
Sampling Rate (GS/s)		**1.5**	3	3	2	10
Resolution (bits)		**12**	12	12	11	12
SNDR@Nyq. (dB)		**58.9**	58.8	56.7	57.3	53.5
ENOB@Nyq. (bits)		**9.49**	9.48	9.13	9.23	8.59
SFDR@Nyq. (dB)		**74.3**	70.7	75.2	69.9	65.0
Total Power (mW)		**102.4**	32.5	46	58.6	750
FoM_W_ † (fJ/conv-step)		**94.8**	15.2	27.5	48.9	194
FoM_S_ # (dB)		**157.6**	165	161.8	159.6	152
Amplifier Calibration	BG Cal. *?	**√**	√	×	×	√
	On-Chip?	**√**	√	×	×	√

*: Background Calibration; †: $FoM_{W}=P/(2_{ENOB}\cdot f_{S})$; #: $FoM_{S}=SNDR+10\log_{10}[f_{s}/(2P)]$.

## Data Availability

The datasets produced and/or analyzed in the present study are available from the corresponding author upon reasonable request.

## Abbreviations

The following abbreviations are used in this manuscript:

ADC	Analog-to-Digital Converter
DNL	Differential Nonlinearity
INL	Integral Nonlinearity
4096-QAM	4096-quadrature amplitude modulation
MDAC	Multiplying Digital-to-Analog Converter
RA	Residue Amplifier
IGE	Interstage Gain Error
SNDR	Signal-to-Noise-and-Distortion Ratio
SFDR	Spurious-Free Dynamic Range
PVT	Process, voltage, and temperature
LMS	Least Mean Squares
DAC	Digital-to-Analog Converter
PN	Pseudorandom Number
RQ-AVS	Rational-Quadratic Adaptive-Variable-Step
AVS	Adaptive-Variable-Step
SRAM	Static Random-Access Memory
SPI	Serial Peripheral Interface
ENOB	Effective Number of Bits
FoM_S_	Schreier Figure of Merit
FoM_W_	Walden Figure of Merit
